# Education-based health inequalities in 18,000 Norwegian couples: the Nord-Trøndelag Health Study (HUNT)

**DOI:** 10.1186/1471-2458-12-998

**Published:** 2012-11-19

**Authors:** Sara Marie Nilsen, Johan Håkon Bjørngaard, Linda Ernstsen, Steinar Krokstad, Steinar Westin

**Affiliations:** 1 Department of Public Health and General Practice, Faculty of Medicine, Norwegian University of Science and Technology, Trondheim, Norway; 2 The Liaison Committee between the Central Norway Regional Health Authority and the Norwegian University of Science and Technology, Trondheim, Norway; 3 Forensic Department and Research Centre Bröset, St. Olav’s University Hospital Trondheim, Trondheim, Norway; 4 Faculty of Nursing, Sør-Trøndelag University College, Trondheim, Norway; 5 HUNT Research Centre, Department of Public Health and General Practice, Faculty of Medicine, Norwegian University of Science and Technology, Levanger, Norway; 6 Levanger Hospital, Nord-Trøndelag Health Trust, Levanger, Norway; 7 Faculty of Medicine, NTNU, Trondheim, NO, 7489, Norway

**Keywords:** Anxiety, Couples, Depression, Education, Family health, Multilevel analysis, Subjective health

## Abstract

**Background:**

Education-based inequalities in health are well established, but they are usually studied from an individual perspective. However, many individuals are part of a couple. We studied education-based health inequalities from the perspective of couples where indicators of health were measured by subjective health, anxiety and depression.

**Methods:**

A sample of 35,980 women and men (17,990 couples) was derived from the Norwegian Nord-Trøndelag Health Study 1995–97 (HUNT 2). Educational data and family identification numbers were obtained from Statistics Norway. The dependent variables were subjective health (four-integer scale), anxiety (21-integer scale) and depression (21-integer scale), which were captured using the Hospital Anxiety and Depression Scale. The dependent variables were rescaled from 0 to 100 where 100 was the worst score. Cross-sectional analyses were performed using two-level linear random effect regression models.

**Results:**

The variance attributable to the couple level was 42% for education, 16% for subjective health, 19% for anxiety and 25% for depression. A one-year increase in education relative to that of one’s partner was associated with an improvement of 0.6 scale points (95% confidence interval = 0.5–0.8) in the subjective health score (within-couple coefficient). A one-year increase in a couple’s average education was associated with an improvement of 1.7 scale points (95% confidence interval = 1.6–1.8) in the subjective health score (between-couple coefficient). There were no education-based differences in the anxiety or depression scores when partners were compared, whereas there were substantial education-based differences between couples in all three outcome measures.

**Conclusions:**

We found considerable clustering of education and health within couples, which highlighted the importance of the family environment. Our results support previous studies that report the mutual effects of spouses on education-based inequalities in health, suggesting that couples develop their socioeconomic position together.

## Background

A social gradient in health status is a major feature of all industrialized countries [1]. Several studies have reported that the gradient varies between women and men, where it is usually less pronounced in women, although this depends on the method used to measure the socioeconomic position [2-8]. However, women and men are often part of a couple. Therefore, it has been argued that characteristics of both individual and partner should be considered in studies of socioeconomic inequalities related to health [2,7,9]. Skalická and Kunst [2] showed that, in addition to a man’s own characteristics, the education of his wife was a strong predictor of his mortality, whereas the husband’s occupation and income were significant predictors of mortality in women. Monden and colleagues [10] showed that education-based gradients in subjective health and smoking were steeper when the education levels of both partners were considered compared with analyses that considered the education of individuals. Jaffe and et al. [3] found that the educational discrepancy between spouses did not affect mortality, whereas the educational levels of both spouses were significant predictors of one’s own mortality.

There is a well-established positive association between education and health [11-15], and several studies have demonstrated that the educational level of one partner affects the health of the other [3,10,16,17]. However, the health differences within couples with different socioeconomic position have not been resolved, and measures of socioeconomic position and health inequalities must still be refined. Separate comparisons of health inequalities in women and men fail to consider that individuals form part of a group, typically a couple, which involve strong mutual effects [18]. Given the well-known tendency to marry partners of equal status [19-21] and the mutual effects of partners, any health inequalities may be amplified by the clustering of similar characteristics in couples.

Significant health similarities have been demonstrated in couples, particularly depression and other mental health problems [22]. Possible explanations for these similarities include assortative mating, shared resources, social control by the spouse and mood convergence [22]. Assortative mating implies that individuals are more likely to marry someone with similar characteristics, attitudes, and behaviours, which can also lead to health concordance. The shared resource hypothesis suggests that a shared environment, financial resources and social networks translate into shared health risks within couples [22,23].

In the present large population study of almost 18,000 couples, we studied: a) the level of clustering related to education, subjective health and anxiety and depression symptoms in couples; and b) education-related differences in health within and between couples.

## Methods

### The Nord-Trøndelag Health Study

The analysis was restricted to married or cohabiting women and men aged >24 years from the Nord-Trøndelag Health Study 1995–97 (HUNT 2). All inhabitants of Nord-Trøndelag County in Norway aged ≥20 years were invited to participate in the health survey [24,25]. In total, 65,600 individuals participated in the overall HUNT 2 study, which constituted 71% of the adult population. The participants completed questionnaires and were screened using a number of health measures. We excluded 384 subjects who participated in the clinical examination but did not complete the questionnaires. We wanted most of the participants to have completed their education, so we excluded 4,061 who were younger than 25 years. We excluded 10,083 unmarried non-cohabiting individuals, 5,670 widowed individuals, 3,675 divorced individuals, 773 judicially separated individuals, four registered partners and 124 individuals who lacked marital status information, which left 40,826 individuals. We excluded 312 individuals from this sample because they lacked educational data, and 4,534 individuals whose partners did not participate in the HUNT 2 study. Thus, the final sample comprised 35,980 individuals, i.e., 17,990 women and 17,990 men. The Norwegian Data Inspectorate and the Regional Committee for Medical Ethics approved the protocols for HUNT 2 and this study. All of the participants provided written consent.

### Health measures

Subjective health was measured by asking: “How is your health at present?” The answer categories were “very good”, “good”, “fair” and “poor”. Subjective health has a strong and consistent association with mortality, and it can be used as an indicator of bodily condition [26]. Anxiety and depressive symptoms were assessed using the Hospital Anxiety and Depression Scale [27]. The symptom scales contain seven items related to anxiety and seven related to depression, and they comprise a well-validated anxiety and depression screening tool for general population samples [28]. The anxiety and depression scales each yielded a total score ranging from 0 to 21, where 21 represented the highest symptom level. We also included those subjects who completed five or six items, and their scores were based on the completed items multiplied by 7/5 or 7/6, respectively. Complete information was provided for all seven items in the anxiety scale by 82% of women and 85% of men, while 92% of both women and men completed all of the depression scale.

Each of the three health measures was rescaled from 0 to 100 to compare the coefficients, where 100 represented the worst health score. Thus, the effects were considered relative to the maximum possible poor health score.

### Education and the family identification number

Educational data were obtained from Statistics Norway; i.e., the number of years each respondent attended school (years of education). The couples were identified based on marriage and cohabitation information in the HUNT 2 study, which was combined with their family identification numbers from the national registers. Residents with the same registered address who were related as spouses, cohabitants, or parents and children were given identical family identification numbers. Cohabitation is common and institutionalized in Scandinavian countries, including Norway [29], and our sample comprised 5% cohabitants and 95% married couples. We used the educational information and family identification numbers at the time of participation in HUNT 2 (1995–97). The HUNT 2 data were linked to the educational data and the family identification number using the 11-digit identity number that is allocated to Norwegian citizens at birth. The identity numbers were removed before the data were made available to the investigators. Education was analyzed as a continuous measure (8–21 years).

### Statistics

We applied an analytical approach that is used widely for modelling clustered data (including data clustered as dyads) to study health differences in couples where the education levels of the partners differed and to determine the education-based gradient in health between couples [18,30-32]. Thus, we used two-level linear random effect regression models [30] to distinguish the individual-based and couple-based variance in health, where Y denoted the health measures (subjective health, anxiety and depression) and X denoted the covariate of interest (education). We used *i* to indicate the couple and *j* to indicate a partner in the *i*th couple. If partners have more similar educational levels than two unrelated individuals, a suitable strategy may be to centre their educational level around the couple’s mean, as suggested by Carlin et al. [31]:

(1) $$Y_{\mathrm{ij}}=\beta_{0}+\beta_{W}\left({Χ}_{\mathrm{ij}}-{\overset{―}{Χ}}_{i}\right)+\beta_{B}{\overset{―}{Χ}}_{i}+\beta GENDER_{\mathrm{ij}}+\beta AGE_{\mathrm{ij}}+U_{i}+e_{\mathrm{ij}}$$

where ${\overset{―}{Χ}}_{i}$ represents the mean value of *X* for couple *i*, the within-couple coefficient ($\beta_{W}\left({Χ}_{\mathrm{ij}}-{\overset{―}{Χ}}_{i}\right)$) represents the health differences between partners per year difference in education and the between-couple coefficient ($\beta_{B}{\overset{―}{Χ}}_{i}$) represents the health differences between couples per year difference in education. Gender and age were also included in the model. Finally, *U*_i_ + *e*_ij_ represent the random component of the model. This model specification allowed us to investigate whether the between-couple coefficient differed from the within-couple coefficient. If the within- and between-couple coefficients were equal, the education-based differences in health between couples would have been consistent with that predicted from the individual levels of education. We also used a model based on an individual’s educational level and the partner’s education level:

(2) Yij=β0+βWΧijo+βBΧijp+βGENDERij+βAGEij+Ui+eij

where (_*o*_*X*_*ij*_) represents the individual’s education level and (_*p*_*X*_*ij*_) is the partner’s educational level. Based on the results from *equation 1*, we used the lincom command in Stata to estimate the health scores of three couples aged 45–54 years with different levels of education.

The degree of clustering was captured using the intra-class correlation (ICC), which reflected the proportion (or percentage when multiplied by 100) of health variance attributable to the differences between couples. The ICC was estimated as the couple-level variance divided by the total variance (*U*_*i*_/*U*_*i*_ + *e*_*ij*_ × 100) [30]. We adjusted for age using 10-year age groups. We also estimated gender-based differences in the education–health association by including an interaction term for gender and education. We also stratified the models by age (<50 years, ≥50 years) and by cohabitation versus married couples. The coefficients were reported with their 95% confidence intervals (95% CIs).

**Figure 1:**
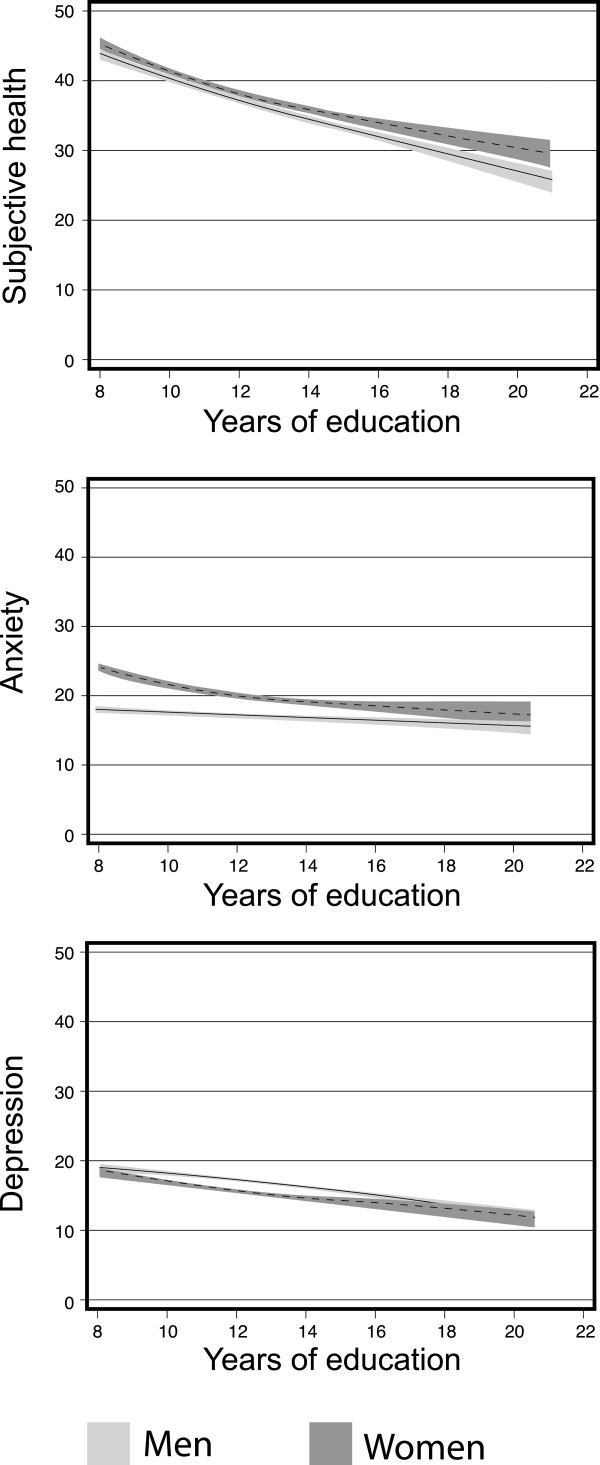
**Age-adjusted subjective health and symptoms of anxiety and depression (scaled from 0 to 100, where 100 represents the worst health score), by years of education in 35,980 women and men aged > 24 years. **The Nord-Trøndelag Health Study 1995–97.

We used cubic splines (the mkspline command in Stata; i.e., three knots where the knot locations were assigned by the statistical software) as a descriptive measure of the associations between education and health outcomes (Figure 1).

## Results

Table 1 shows summary information for our sample of 17,990 couples; i.e., 17,990 women and 17,990 men. The mean age was 51 years (standard deviation (SD) = 14 years) for women and 54 years (SD = 14 years) for men. The mean length of education was 12 years (SD = 3 years). Of the individuals in our sample, 24% had primary education, 44% had secondary education and 32% had tertiary education. Among the total population of Norway at the time of the survey, 27% had primary education, 53% had secondary education and 20% had tertiary education (http://www.ssb.no). The mean scaled subjective health was 39 scale points (SD = 22–23) for both women and men. The mean anxiety scores were 22 scale points (SD = 16) for women and 18 scale points (SD = 14) for men. The corresponding figures for the depression symptom scale were 16 (SD = 14) and 18 (SD = 14), respectively. An individual’s education level was associated with a lower morbidity score for all three health measures (Figure 1).

**Table 1:** Characteristics for 35,980 married or cohabiting women and men >24 years

	**Women**		**Men**	
**Characteristics**	**Mean**	**(SD)**	**Mean**	**(SD)**
Age in years	51	14	54	14
Education in years	12	3	12	3
Subjective health^a^	39	23	39	22
Missing (%)	1		1	
Anxiety^a^	22	16	18	14
Missing (%)	5		4	
Depression^a^	16	14	18	14
Missing (%)	3		3	
Education	N	(%)	N	(%)
*Primary*	4,398	(25)	4,149	(23)
*Secondary*	8,662	(48)	7,314	(41)
*Tertiary*	4,930	(27)	6,527	(36)
Total	17,990	(100)	17,990	(100)

The Nord-Trøndelag Health Study 1995–97.

^a ^Scaled from 0–100 where 100 represent the worst health score.

Based on the results of the two-level linear random effect regression model where education was the dependent variable, the ICC for couples for education was 42% (results not shown), which reflected the percentage of variance attributable to the couple level or, alternatively, the degree of within-couple clustering for education. The ICCs were 16% for subjective health, 19% for anxiety and 25% for depression (Model 3, Tables 2 and 3).

**Table 2:** **Two-level linear random effect regression models for the association between education in years and subjective health**^**a **^**in 17,990 couples**

	**Subjective health**					
	**Model 1**		**Model 2**		**Model 3**	
	**β**	**(95% CI)**	**β**	**(95% CI)**	**β**	**(95% CI)**
Intercept	44		49		49	
Women compared to men	1.2	(0.8, 1.6)	1.4	(1.0, 1.8)	1.4	(1.0, 1.8)
Age						
25-34	ref		ref		ref	
35-44	3.7	(2.9, 4.6)	3.7	(2.9, 4.5)	3.7	(2.9, 4.5)
45-54	9.2	(8.3, 10.0)	8.9	(8.0, 9.7)	8.9	(8.0, 9.7)
55-64	14.3	(13.4, 15.2)	13.6	(12.7, 14.5)	13.6	(12.7, 14.5)
>64	17.1	(16.2, 18.0)	16.1	(15.1, 17.0)	16.1	(15.1, 17.0)
*Education*						
Own education	−1.3	(−1.4, -1.3)	−1.2	(−1.3, -1.1)		
Partner education			−0.5	(−0.6, -0.4)		
Within couple^b^					−0.6	(−0.8, -0.5)
Between couple^c^					−1.7	(−1.8, -1.6)
Individual level variance	367		365		365	
Couple level variance	66		67		67	
Intra class correlation (%)^d^	15		16		16	

The Nord-Trøndelag Health Study 1995–97, women and men >24 years.

^a ^Scaled from 0–100 where 100 represent the worst health score.

^b ^The within couple coefficient gives the expected change in health score for one-year education change in the difference between the individual education and the couple average education, holding the latter constant.

^c ^The between couple coefficient gives the expected change in health score for one-year education change in the couple average education, while holding the individual deviation from the average constant.

^d ^Couple level variance divided by the total variance, multiplied by 100.

**Table 3:** Two-level linear random effect regression models for the association between education in years and symptoms of anxiety and depression in 17,990 couples

	**Anxiety**^**a**^						**Depression**^**a**^					
	**Model 1**		**Model 2**		**Model 3**		**Model 1**		**Model 2**		**Model 3**	
	**β**	**(95% CI)**	**β**	**(95% CI)**	**β**	**(95% CI)**	**β**	**(95% CI)**	**β**	**(95% CI)**	**β**	**(95% CI)**
Intercept	23		25		25		19		22		22	
Women compared to men	3.5	(3.2, 3.8)	3.5	(3.2, 3.8)	3.5	(3.2, 3.8)	−1.1	(−1.4, -0.9)	−1.1	(−1.3, -0.8)	−1.0	(−1.3, -0.8)
Age												
25-34	ref		ref		ref		ref		ref		ref	
35-44	0.2	(−0.4, 0.8)	0.2	(−0.4, 0.8)	0.2	(−0.4, 0.8)	2.1	(1.5, 2.7)	2.1	(1.5, 2.6)	2.1	(1.5, 2.6)
45-54	0.2	(−0.4, 0.8)	0.1	(−0.6, 0.7)	0.1	(−0.6, 0.7)	4.6	(4.0, 5.2)	4.4	(3.8, 5.0)	4.4	(3.8, 5.0)
55-64	−0.3	(−0.9, 0.4)	−0.5	(−1.2, 0.1)	−0.5	(−1.2, 0.1)	6.0	(5.4, 6.6)	5.6	(5.0, 6.2)	5.6	(5.0, 6.2)
>64	−3.0	(−3.7, -2.3)	−3.4	(−4.1, -2.8)	−3.4	(−4.1, -2.7)	6.6	(6.0, 7.2)	6.0	(5.3, 6.6)	6.0	(5.3, 6.6)
*Education*												
Own education	−0.4	(−0.4, -0.3)	−0.3	(−0.4, -0.2)			−0.5	(−0.6, -0.4)	−0.4	(−0.5, -0.4)		
Partner education			−0.2	(−0.3, -0.2)					−0.3	(−0.4, -0.2)		
Within couple^b^					−0.1	(−0.2, 0.0)					−0.1	(−0.2, 0.0)
Between couple^c^					−0.5	(−0.6, -0.5)					−0.7	(−0.8, -0.6)
Individual level variance	191		191		191		147		147		147	
Couple level variance	43		44		44		50		50		50	
Intra class correlation (%)^d^	18		19		19		25		25		25	

The Nord-Trøndelag Health Study 1995–97, women and men >24 years.

^a ^Scaled from 0–100 where 100 represent the worst health score.

^b ^The within couple coefficient gives the expected change in health score for one-year education change in the difference between the individual education and the couple average education, holding the latter constant.

^c ^The between couple coefficient gives the expected change in health score for one-year education change in the couple average education, while holding the individual deviation from the average constant.

^d ^Couple level variance divided by the total variance, multiplied by 100.

Model 1 (Tables 2 and 3) shows the association between an individual’s education level and subjective health, anxiety symptom score and depression symptom score, after adjustments for gender and age. Model 2 (Tables 2 and 3) shows the association between an individual’s education, partner’s education and the health outcomes. Model 3 (Tables 2 and 3) included the mean educational level of the couple.

**Figure 2:**
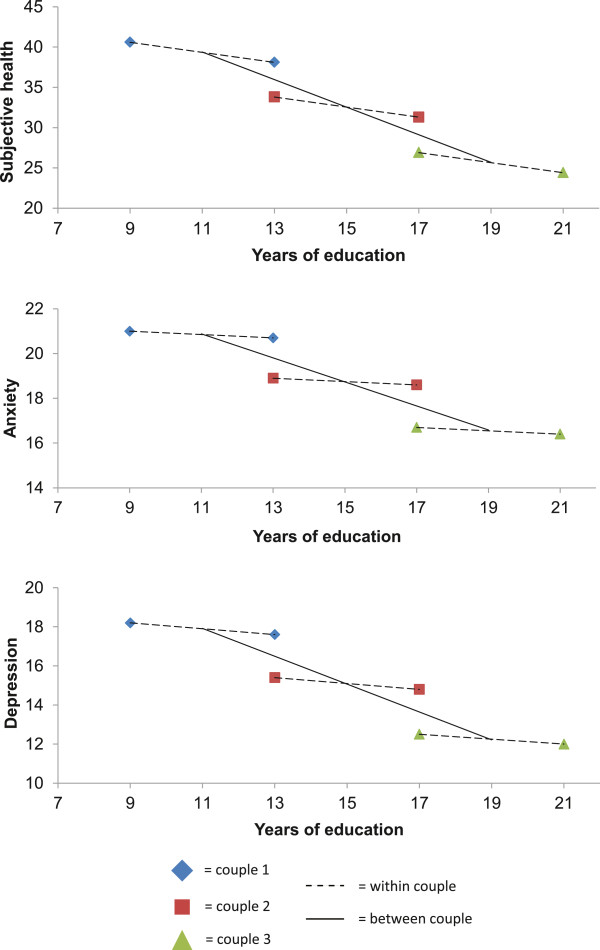
**Expected scores for subjective health and symptoms of anxiety and depression (scaled from 0 to 100, where 100 represents the worst health score) within and between three couples at the age of 45–54 years with different levels of education. **Estimates are based on Tables 2 and 3, Model 3.

In Model 1 (Table 2), a one-year increase in an individual’s educational level was associated with a subjective health improvement of 1.3 scale points (95% CI 1.3–1.4). When the partner’s education was kept constant in Model 2, a one-year increase in the individual’s educational level was associated with a subjective health improvement of 1.2 scale points (95% CI 1.1–1.3). When the individual’s own education was kept constant, a one-year increase in the partner’s education was associated with a subjective health improvement of 0.5 scale points (95% CI 0.4–0.6). In Model 3, a one-year increase in the educational level relative to one’s partner was associated with a subjective health score improvement of 0.6 scale points (95% CI 0.5–0.8; within-couple coefficient). A one-year increase in the average educational level of a couple was associated with a subjective health score improvement of 1.7 scale points (95% CI 1.6–1.8; between-couple coefficient). In Model 1 (Table 3), a one-year increase in an individual’s educational level was associated with anxiety and depression symptom score improvements of 0.4 scale points (95% CI 0.3–0.4) and 0.5 scale points (95% CI 0.4–0.6), respectively. When the partner’s education was kept constant in Model 2, a one-year increase in an individual’s educational level was associated with anxiety and depression symptom score improvements of 0.3 scale points (95% CI 0.2–0.4) and 0.4 scale points (95% CI 0.4–0.5), respectively. The corresponding figures for the partner’s educational levels were 0.2 scale points (95% CI 0.2–0.3) for the anxiety symptom score and 0.3 scale points (95% CI 0.2–0.4) for the depression symptom score, when the individual’s educational level was kept constant. In Model 3, a one-year increase in the educational level relative to one’s partner was associated with an improvement of 0.1 scale points (95% CI 0.0–0.2) in the anxiety symptom score and an improvement of 0.1 scale points (95% CI 0.0–0.3) in the depression symptom score. A one-year increase in the couple’s average educational level was associated with an improvement of 0.5 scale points (95% CI 0.5–0.6) in the anxiety symptom score and an improvement of 0.7 scale points (95% CI 0.6–0.8) in the depression symptom score. The within- and between-couple coefficients differed significantly for all outcome measures (*P* < 0.01). The within- and between-couple associations are illustrated in Figure 2, which shows the expected health scores for three couples with different educational levels (couple 1 with 9 and 13 years of education, couple 2 with 13 and 17 years of education and couple 3 with 17 and 21 years of education) within and between the couples. The confidence intervals of the estimates in Figure 2 were too narrow to be displayed (<2 scale points).

We found no evidence for an interaction between gender and the education level, with the exception of the anxiety symptom scores, where an increase in a couple’s educational level improved the anxiety score more for women than men; i.e., an improvement of 0.8 scale points (95% CI 0.7–1.0) for a one-year increase in educational level for women and a corresponding improvement of 0.2 scale points (95% CI 0.1–0.3) for men. For women, there was also an improvement of 0.3 scale points (95% CI 0.1–0.5) in the anxiety symptom score when the partner had a lower educational level. We performed the analyses of married and cohabiting couples separately, and the results were similar to those presented above. This was also the case for an additional analysis of individuals <50 years, ≥50 years.

There were no differences in the subjective health of men and women in Model 1. The subjective health of the oldest age group (> 64 years) was 16.1 scale points (95% CI 15.1–17.0) worse than that of the youngest age group (25–34 years) in Model 3.

The anxiety symptom score of women was 3.5 scale points (95% CI 3.2–3.8) higher than that of men (Model 3). The anxiety symptom score of the oldest age group (> 64 years) was 3.4 scale points (95% CI 3.8–3.2) lower than that of the youngest age group in Model 3.

In Model 3, the depression symptom score for women was 1.1 scale points (95% CI 0.9–1.4) lower than that of men, and age was linearly associated with an increasing depression symptom score.

## Discussion

We found a high degree of clustering in the educational level, subjective health, anxiety and depression symptom scores of couples. More education was associated with better health. The education-based differences in health were less pronounced within couples, but combining the information from both partners detected substantial gradients for all three health measures.

We detected a clear trend where partners had similar levels of education, which is consistent with the findings of other recent studies [3,10]. We also found smaller education-based differences in health within couples compared with our models that did not consider educational clustering within couples. However, combining the educational levels of couples detected a strong between-couples educational gradient for all health measures. These results suggest that the educational level can be used as a couple-level socioeconomic status measure, as well as an individual characteristic. Health inequality studies based on individual incomes are often considered weak because the household income is generally considered to be a more accurate measure, particularly for women [33]. Similarly, the educational level of a family/couple might better reflect individual educational capital for women and men. Like income, it is possible that many couples view their education as a common investment, where longer education is a shared resource for the family. However, health inequalities may be amplified between couples because of the clustering of characteristics within couples [19-21]. It is also more likely that the health or lifestyle of a couple will follow a “low-education pattern” if a less-educated person has a less-educated partner, compared with a situation where one member of the couple is more highly educated [10,34].

There is evidence of socioeconomic inequalities in depression [35,36]. We found no education-based differences in the anxiety and depression symptom scores within couples. However, we found substantial socioeconomic inequalities in common mental health problems when measured at the couple level. Few studies have considered the clustering effects of couples on mental health in a multilevel framework. A recent review [22] concluded that there was strong evidence of concordant mental health and health behaviour in couples, which highlighted the need to employ new methodological techniques such as multilevel modelling to account for this dependency in studies.

A few other studies have indicated substantial health clustering at the household level [37-39], particularly for depression [22]. A British study [37] found that the ICC for subjective health was 20%, which was little different from our result of 16%. However, it is interesting to note that the estimates for health clustering in our study could not be captured by adjusting for the educational level. Thus, our results did not indicate that health clustering in couples could be attributed to the tendency for partners to have similar educational levels.

Previous studies have suggested that socioeconomic gradients are less pronounced for women than men [7,9], and a Dutch study [10] found that women were more affected by the education of their partners than men in terms of their subjective health and excessive alcohol consumption. However, our results did not suggest a statistical interaction between education and gender, although an increase in the couple’s educational level was accompanied by a slightly higher reduction in the anxiety score for women than for men, while there was a tendency for the anxiety score to be lower in women when their partners had lower educational level.

We used education as an indicator of socioeconomic position because it has important effects on work and income, which makes it the key to an individual’s position in the stratification system [40]. Education is generally available to both women and men, and it is less vulnerable to negative health selection than other factors such as occupational class or income. We could not draw causal conclusions based on this cross-sectional study, but individuals are probably affected by the education of their partners via mechanisms similar to those that influence the health gradient relative to their own education, including their material circumstances, psychosocial environment, working life and lifestyle [10].

### Strengths and limitations

The present study was based on a large unselected population sample. Linking the data to educational level and family identification numbers from national registers ensured that our measurements were valid and reliable. However, a non-participation study of the HUNT 2 population [41] revealed higher drop-out rates for individuals with high alcohol consumption, poor mental health and/or a smoking habit. Lifestyle factors and low socioeconomic position are associated with non-participation in epidemiological studies [42], so we may have underestimated the levels of poor subjective health, anxiety, depression and educational inequalities. Generalizability from the Nord-Trøndelag population to the overall Norwegian population is considered good because the geography and demography of the region are representative, and it has average socioeconomic mortality [24]. Thus, the database (HUNT 2, 1995–97) should be suitable for a conceptual study, rather than a prevalence report. The morbidity changes over time in populations, but there have been no dramatic changes in this population in recent years.

Couples are not static units, and the marriage statistics are affected by divorce, remarriage, the increasing age of first marriage, the increasing numbers that never marry and increasing levels of cohabitation [22]. Furthermore, the variance attributed to couples may be explained by individual-level characteristics that were not included in our model. However, others have reported similarities within couples in terms of their subjective health status, even after a number of individual characteristics were considered [37]. Finally, small group sizes arise by necessity in studies of couples. However, a recent study [43] concluded that the estimates of multilevel models are generally not affected by small group sizes if the number of groups is large.

## Conclusions

We found high levels of clustering among couples with respect to education, subjective health, anxiety and depression symptom scores. Our results support previous studies that report the mutual effects of spouses on education-based inequalities in health, suggesting that couples develop their socioeconomic position together. Thus, research and public health interventions should pay more attention to the importance of households, families and couples.

## Competing interests

The authors declare that they have no competing interests.

## Authors’ contributions

SMN processed the data and wrote the first draft of the manuscript. JHB and SMN conducted the statistical analyses and interpreted the data. LE, SK, and SW participated in the design of the study and helped to write the manuscript. All authors have critically revised the manuscript, and read and approved the final version.

## Pre-publication history

The pre-publication history for this paper can be accessed here:

http://www.biomedcentral.com/1471-2458/12/998/prepub
